# Hepatitis E virus genome plasticity: a marker of chronicity and therapy failure?

**DOI:** 10.1038/s42003-026-11055-5

**Published:** 2026-10-22

**Authors:** Saskia Janshoff, Leyla Sirkinti, Maximilian K. Nocke, Theresa S. Bechtel, André Gömer, Yannick Brüggemann, Eike Steinmann, Richard J. P. Brown, Daniel Todt

**Affiliations:** 1 Ruhr University Bochum, Medical Faculty, Department of Translational and Computational Infection Research (TRACiR), Bochum, Germany; 2 Ruhr University Bochum, Medical Faculty, Department of Molecular und Medical Virology, Bochum, Germany; 3 Institut für Laboratoriums- und Transfusionsmedizin, Herz- und Diabeteszentrum Nordrhein-Westfalen, Universitätsklinik der Ruhr-Universität Bochum, Bad Oeynhausen, Germany; 4 Hepatitis E Virus Research Hub (HepE-Hub), Bochum, Germany; 5 European Virus Bioinformatics Center (EVBC), Jena, Germany

**Keywords:** Virus-host interactions, Viral evolution

## Abstract

Hepatitis E virus (HEV) is a globally distributed zoonotic pathogen, spread by contaminated food and blood products, and a leading cause of acute viral hepatitis. Recent data indicate that the HEV genome contains a highly heterogeneous hypervariable region (HVR) that can tolerate extensive genomic rearrangements, especially among immunocompromised patients. These rearrangements can increase HEV replication in tissue-culture and have further been observed in HEV-infected patients. Insertions comprise either duplications of virus-derived sequences, or alternatively, host-derived sequence fragments and exhibit enrichment of specific amino acids and functional motifs. However, the molecular mechanisms driving these insertions, as well as their functional significance, remain largely unexplored. Given that sequences not required for infectivity or replication are typically rapidly eliminated in small RNA viruses such as HEV, the persistence of HVR insertions suggests a potential biological role in HEV replication and/or pathogenesis. This review summarizes our current understanding of the role of HVR insertions during HEV infection, as well as the potential mechanisms underlying these insertions. We further discuss the factors driving the selection of genomic insertions and speculate on their functional role by analyzing over 650 publicly available HEV genotype 3 genome sequences and systematically cataloging identified insertions.

## Introduction

Hepatitis E virus (HEV) is a major cause of acute viral hepatitis globally. In 2021, the World Health Organization (WHO) estimated 19.47 million cases of acute HEV, resulting in 3450 deaths^1,2^. HEV is classified within the *Hepeviridae* family and the *Paslahepevirus* genus. Based on phylogenetic analysis, globally sampled HEV can be subdivided into eight distinct genotypes (HEV1-HEV8), with HEV1-4 and HEV7 able to infect humans^3–5^. Three open reading frames (ORF1-3) are encoded by the 7.2 kilobase pair (kbp) HEV genome, which is a single-stranded positive-sense RNA molecule ( + ssRNA)^6^. HEV-3 is the most common genotype in industrialized countries^7^ and is transmitted through zoonotic routes^8^, including contact with infected swine, consumption of contaminated meat, environmental exposure^9–11^, and additionally via contaminated blood products^12^. HEV-3 is endemic in developed countries^13^, where it often causes an asymptomatic course of infection. In contrast to that, HEV-3 represents a considerable risk for immunocompromised patients, where infection can transition to chronicity and potentially result in progression to severe liver disease^14^. Current guidelines recommend ribavirin (RBV) as the preferred therapy. In selected liver transplant recipients, pegylated interferon-alpha (PEG-IFN-α) may be considered; however, it is avoided in other solid-organ transplant (SOT) recipients due to the risk of graft rejection (EASL)^15^. Nevertheless, roughly 20% of RBV-treated patients experience treatment failure, and a prophylactic vaccine is not broadly available. Therefore, HEV poses a challenge in clinical practice, because iatrogenic transmission can occur via two independent routes: blood transfusion and solid-organ transplantation^16^. Achieving a more detailed understanding of the progression of disease in chronic HEV-3 infection and determining the underlying drivers of the transition from acute to chronic infection is imperative for the development of effective clinical management strategies. Previous studies have shown that intra-host variability in HEV genomes (ORF1 HVR, macrodomain, and ORF2) during the acute phase of infection is associated with progression to chronicity^17,18^. In addition, length polymorphisms in the hypervariable region (HVR) of ORF1, i.e., insertions of both host- and virus-derived gene snippets of considerable length (see below) have been detected in ORF1 HVR^18–22^. To date, there is no clear evidence that detection of such insertions during acute infection can be predictive of progression to chronicity. However, these rearrangements may represent a minor subpopulation within the viral quasispecies undetectable by conventional Sanger sequencing, which can only be identified using high-throughput sequencing (HTS) approaches^21^. The ratio of viral genomes within the viral population presenting these length polymorphisms can vary over time. Furthermore, these rearrangements are exclusively focused in the HVR within ORF1 where certain insertions have been associated with increased viral fitness in tissue culture^22–26^. Together, these data suggest that the HVR plays an important role in HEV replication and pathogenesis and may represent a determinant associated with the transition to chronicity under immunosuppression. The molecular mechanisms that give rise to these “self-duplicating” and/or “host-sequence snatching” events have yet to be elucidated. Furthermore, there is a lack of surveillance and efficient identification algorithms to enable early detection and monitoring of genomic rearrangements in HEV.

In this review, we first give a brief overview of HEV clinical manifestations and molecular virology, discussing the genomic flexibility observed in HEV HVR in both chronic and acute infection. We then integrate publicly available viral sequence data and host transcriptome datasets to catalog HVR insertion sites, illuminating potential modes of action for the integration of both viral and host sequences and yielding novel mechanistic insights. Finally, we assess technology platforms best suited for analysis of HEV HVR diversity in patients and their clinical utility.

### Clinical perspective on hepatitis E

The clinical manifestations associated with HEV infection range from asymptomatic to acute liver failure. Symptoms of HEV infection are similar to those of other forms of acute hepatitis, including jaundice, fatigue, abdominal pain, nausea, and elevated liver enzymes^16^. Acute hepatitis is typically self-limiting in immunocompetent individuals, with a low mortality rate and an incubation period of 2–10 weeks^1,27^. Especially in the context of pregnancy, HEV-1 infections have been reported with a higher risk of severe outcomes^28^, and HEV-1-2 infections are associated with a higher ratio of symptomatic cases ( ~ 16%) compared to HEV-3-4 ( ~ 2–3%)^29–31^. Beyond genotype-level differences, accumulating evidence indicates that HEV-3 subtype diversity also shapes the severity of acute infection, although a comparable link to chronic infection has not been established. HEV-3 can be subdivided phylogenetically into two major clades. Clade 1 includes HEV-3 efg, while clade 2 is comprised of HEV-3 abchijklmno^32–37^. Clade 1 strains have repeatedly been associated with a more severe clinical phenotype than clade 2 strains. UK blood donors carrying clade 1 virus showed higher viral loads and more frequent symptoms than those infected with predominantly clade 2 virus^38^. A Belgian cohort reported 1.7-fold higher hospitalization risk for clade 1 versus clade 2 infection, with subtype 3c specifically carrying the lowest risk^39^, and a German surveillance cohort of 1,656 sequenced cases confirmed that jaundice, fever, hospitalization, and death were all significantly more frequent among clade-1-infected patients^40^. In contrast, no HEV-3 subtype has been convincingly linked to an increased risk of progression to chronicity. The subtypes most frequently reported in chronically infected immunocompromised and transplant patients (HEV-3c, e, f, and i) largely mirror those already dominant in general circulation within the same regions. Dedicated virological comparisons have so far failed to identify a subtype-specific determinant of viral persistence^41^. Between 50 – 60% of immunocompromised patients infected with HEV progress into chronicity, potentially leading to liver fibrosis and cirrhosis^14,42–44^. Chronic HEV infection was first reported in liver and kidney transplant patients^14,45^ and is characterized by the persistence of HEV RNA in both the blood and stool for a minimum of three months. Currently, no cases of chronic HEV-1 or HEV-2 have been reported. While chronic HEV infections are primarily associated with SOT recipients, the risk extends to other immunocompromised patients^46–49^.

The vast majority of HEV-infected individuals experience asymptomatic and/or acute infections, ending in seroconversion and viral clearance. In a small number of rare cases in SOT recipients, spontaneous viral clearance was observed without treatment 3-6 months after first detection^44^. However, prompt detection of an ongoing infection is important to enable initiation of antiviral treatment to reduce the chances of severe liver damage and progression to chronicity^15,50^. At present, there are no specific HEV antiviral treatments available, and the first approach after diagnosis of HEV is reduction of immunosuppressive medication in combination with treatment with either pegylated interferon-alpha (PEG-IFN-α) or off-label antiviral drugs, predominantly RBV^15,16,51–53^. PEG-IFN-α is generally contraindicated in SOT recipients, except selected liver-transplant recipients, due to the risk of graft rejection. It may be considered only in selected liver-transplant recipients under close monitoring^54^. In contrast, clinical trials have demonstrated the partial efficacy of RBV in treating chronic HEV infections, although monitoring is required in patients with renal insufficiency and contraindicated in pregnancy. A multicenter retrospective study has demonstrated the efficacy of RBV in 59 SOT patients, achieving a sustained virological response (SVR) in 78%^50^. If patients are not suitable for RBV therapy or are unable to achieve an SVR, sofosbuvir (SOF) has been evaluated as an alternative therapeutic option. SOF is a nucleoside analog and a licensed treatment for chronic hepatitis C virus (HCV). SOF inhibits HEV replication in vitro and has an additive effect in combination with RBV^55^. However, published reports on the successful treatment of chronic HEV infection with SOF are inconsistent^56–65^. A recent pilot clinical trial failed to demonstrate SVR in any of the study participants^66^. Consequently, clinicians are encountering increasing numbers of cases where no SVR can be achieved^66–68^. Recent studies have identified substitutions in the HEV polymerase that are associated with altered replication capacity and reduced susceptibility to antiviral agents in vitro^69–72^. Unlike classical chain-terminating nucleos(t)ide analogs, RBV has been shown to inhibit HEV replication in vitro by depleting cellular GTP pools^53^. Additionally, RBV acts as a mutagen, increasing the viral mutation rate and potentially driving error catastrophe/lethal mutagenesis, a mechanism that has also been described for other RNA viruses^70,73–76^. Other modes of action of RBV may be important in vivo, since the inhibition of inosine-5′-monophosphate dehydrogenase (IMPDH) by mycophenolic acid does not consistently prevent chronicity in SOT patients^77^. In this context, several HEV RdRp substitutions (e.g., Y1320H, K1383N and G1634R) have been identified in patients and associated with enhanced replication capacity, while RBV sensitivity remained unaltered in vitro^69,70,78,79^. In contrast, SOF, a known HCV inhibitor that has been tested in a clinical pilot trial against HEV^66^. Under SOF exposure, the RdRp substitution A1343V has been observed to emerge in the HEV viral population in vivo, reducing SOF sensitivity in vitro^71,72^.

For viruses other than HEV, the role of single-nucleotide variants (SNVs) in antiviral therapy failure has been well established. SNVs can confer an evolutionary advantage by enhancing viral replication and immune evasion and, by analogy, may similarly contribute to viral persistence and the progression of HEV to a chronic state. As with SNVs, insertions and deletions in the HVR have been observed in HEV-infected patients^20–22^ and may affect replication fitness and influence disease progression and the therapy outcome. However, the contribution of genomic insertions to HEV persistence and disease progression has received little attention and remains largely unexplored.

### HEV genomic architecture and replication cycle

Previously, the lack of an HEV cell-culture system limited our understanding of HEV’s replication cycle. However, recent developments in this field have illuminated many aspects of HEV’s biology, including the identification of multiple co-opted host factors hijacked by the virus to facilitate its spread^80–84^. Two key laboratory clones, p6 Kernow^78,85^ and 47832c^86^, have been instrumental in these discoveries due to their efficient replication in cell culture, driven by specific genetic insertions that enhance viral stability and growth in vitro^70,87^. These strains are invaluable for studying HEV pathogenesis, host interactions, and preclinical testing of potential therapeutic interventions. Indeed, these tools have enabled the identification of promising antivirals targeting both the virus and the host^88^. Despite these recent advances, many areas of HEV biology remain poorly defined, although the HEV genome and its organization are relatively well-characterized^6,89^. The 7.2 kb HEV genome is capped at the 5´ end with a 7-methylguanine followed by a 5′ untranslated region (UTR), three ORFs, a 3′UTR, and is polyadenylated at the 3´end. Consequently, it resembles a host mRNA, allowing it to be translated by ribosomes^90–92^. ORF1 is the non-structural domain responsible for virus replication and subgenomic RNA synthesis^93^. ORF2 encodes the viral capsid protein, which assembles to encapsulate the viral RNA and is essential for virus entry into host cells and the target for neutralizing antibody responses^94,95^. ORF3 is involved in the release of infectious virions from cells, but its role in HEV entry or assembly remains unclear^96,97^.

After virus entry, the HEV +sense RNA genome serves directly as a template for ORF1 polyprotein translation by host ribosomes^98,99^. While the proteins encoded by ORF1 are translated from the full-length genomic RNA, the ORF2 and ORF3 proteins are translated from subgenomic RNA (sgRNAs). The HEV bicistronic/second subgenomic unit is 2.2 kb in size, capped with 7-methylguanine at the 5’ end, and responsible for both methionine translation initiation events required for the synthesis of ORF2 and ORF3^100^. A cis-active element located at the junction region between ORF1 and ORF3 controls subgenome expression, facilitating ORF2 and ORF3 translation^89,101,102^.

**Fig. 1:**
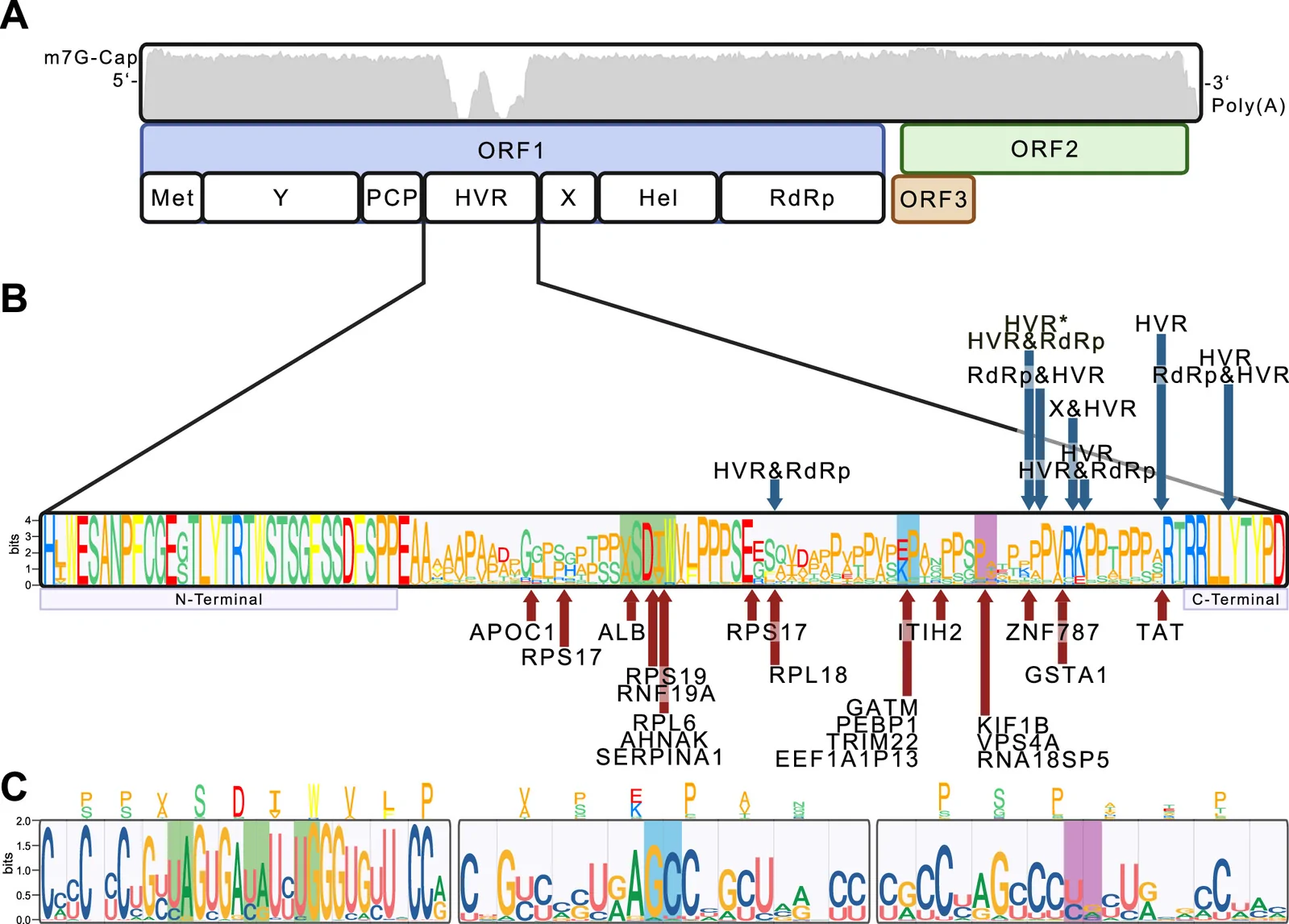
HVR backbone diversity and hotspots for virus-host insertions. **A** The HEV genome consists of single-stranded positive-sense RNA ( + ssRNA), ~7.2 kbp in length, with a 5′ 7-methylguanine cap (m7G-Cap) and a 3′ poly(A) tail. ORF1 encodes non-structural proteins, including methyltransferase (Met), Y domain, papain-like cysteine protease (PCP), HVR, X domain, RNA helicase (Hel), and RNA-dependent RNA polymerase (RdRp), while ORF2 and ORF3 encode the capsid and a multifunctional protein, respectively. Conservation analysis was performed on 671 full-genome sequences (NCBI) using ClustalW^155^ to generate a multiple sequence alignment (MSA), with levels of conservation calculated and plotted in Python v3.12/ R v4.3. **B** From the full-length MSA, the HVR region was extracted, translated to amino acid, and further trimmed to 666 isolates, removing sequences with insufficient coverage. Additional trimming was performed to remove virus-host inserts and duplications to reconstruct the native HVR backbone. Anchoring the alignment to its conserved flanking regions facilitated improved alignment of divergent subgenotypes. Sequence logo representation visualizes conserved positions, length discrepancies (indels), and amino acid variability in the HVR alignment. Arrows indicate insertion hotspots for HEV-derived (blue), and host-derived (red) sequences, with the three most frequent host-insertion sites highlighted (green, blue, purple). Asterisks indicate genotype 3f-specific duplications found in both swine and human samples. **C** Nucleotide-level sequence logos of the top three host-insertion sites, with the insertion positions marked between the two flanking bases (green, blue, purple), together with the corresponding amino acid sequence above. *Parts of figure* c*reated in BioRender. Nocke, M. (2026)* https://BioRender.com/4xs7ch0.

ORF1 is around 5 kb in size and encodes a large non-structural protein^91^ which contains all the necessary components required for HEV genome replication. These include a methyltransferase (Met) domain, a Y domain, a papain-like cysteine protease (PCP) domain, a hypervariable region (HVR), an X domain, an RNA helicase domain, as well as an RNA-dependent RNA polymerase (RdRp)^103,104^ (Fig. 1A). It is currently unknown whether the ORF1-encoded protein is proteolytically processed to release its constituent domains so that they can perform their various functions independently, or whether these domains function together simultaneously in an uncleaved multidomain protein^105^. Underlining the knowledge gap, recent Alphafold2 predictions propose alternative domain usage. Instead of a protease function, the region upstream of the HVR is thought to bind divalent metal ions and contain a fatty acid binding domain (FABD)-like domain, with the Met and the Y domains acting together as one single functional domain^106–108^.

The HVR, also referred to as the polyproline-rich region (PPR)^25^, exhibits significant variability both within and between HEV genotypes (Fig. 1). Indeed, HVR amino acid residue identity differs by up to 71% between HEV genotypes 1-8, with HVR length ranging from 107 to 172 residues. Zoonotic HEV genotypes exhibit greater HVR diversity, particularly HEV-3, which stands out due to its high proline content and up to 41% variation in amino acid identity^25,109^. The HVR consists of conserved N- and C-termini flanking a highly variable central domain. Importantly, HEV HVR diversity may play a role during adaptation to new host species^99^ and disease progression in HEV-3-infected immunocompromised patients. Collectively, these characteristics help explain the patterns of HVR diversity observed in acute and chronic HEV infection. The HVR is enriched for genomic mutations, insertions, duplications, and deletions, leading to significant differences in amino acid length^20–22,110^. It maintains amino acid sequence plasticity not solely via accumulation of non-synonymous substitutions, but primarily via the integration of exogenous sequence fragments. Interestingly, these insertions comprise genomic snippets of both viral and host origin^20–22,85,86,111–113^ (Table 1). Studies focusing on immunocompromised patients with persistent HEV infections have documented the accumulation of insertions in HVR over time, and the integration of certain host-derived sequences in HVR has been shown to enhance HEV replication capacity and viral fitness in tissue culture^22,26^.

**Table 1:** Comprehensive Overview of Genomic Rearrangements of HEV

NCBI Access	NCBI Protein	Type of Arrangement	Size of Arrangement (n)	Reference	Name of the Recombinant Variant
**OL469135.1**	ULK74019.1	deletion	24 bp deletion	Biedermann, Liver Int., 2023	19-0142
**OL469136.1**	ULK74020.1	deletion	45 bp deletion	Biedermann, Liver Int., 2023	19-0151
**OL469137.1**	ULK74021.1	deletion	6 bp deletion	Biedermann, Liver Int., 2023	19-0157
**OL469138.1**	ULK74022.1	deletion	15 bp deletion	Biedermann, Liver Int., 2023	19-0174
**OL469139.1**	ULK74023.1	HVR insertion	First HVR insertion: 29 bp+Second HVR insertion: 127 bp	Biedermann, Liver Int., 2023	19-0144
**OL469140.1**	ULK74024.1	RdRp/HVR insertion	RdRp insertion: 37 bp+HVR insertion: 113 bp	Biedermann, Liver Int., 2023	19-0144
**OL469141.1**	ULK74025.1	HVR insertion	First HVR insertion: 28 bp+Second HVR insertion: 68 bp	Biedermann, Liver Int., 2023	19-0174
**OL469142.1**	ULK74026.1	deletion/HVR insertion	15 bp deletion+First HVR insertion: 28 bp+Second HVR insertion: 68 bp	Biedermann, Liver Int., 2023	19-0174
**OL469143.1**	ULK74027.1	RPL18 insertion	147 bp insertion	Biedermann, Liver Int., 2023	19-0195
**OL469144.1**	ULK74028.1	AHNAK insertion	138 bp insertion	Biedermann, Liver Int., 2023	19-0195
**OL469145.1**	ULK74029.1	RdRp/HVR insertion	RdRp insertion: 81 bp+HVR insertion: 81 bp	Biedermann, Liver Int., 2023	19-0195
**OL469146.1**	LK74030.1	GSTA1 insertion	138 bp insertion	Biedermann, Liver Int., 2023	19-0196
**OL469147.1**	ULK74031.1	deletion/GSTA1 insertion	Deletion: 9 bp + Insertion: 138 bp	Biedermann, Liver Int., 2023	19-0196
**OL469148.1**	ULK74032.1	RdRp/No Match/HVR insertion	RdRp insertion: 38 bp + NoMatch insertion: 15 bp + HVR insertion: 79 bp	Biedermann, Liver Int., 2023	19-0196
**MW837253.1**	UKH47156.1	AHNAK insertion	138 bp insertion	Papp, Sci Rep, 2022	17-0421
**MW837244.1**	UKH47145.1	AHNAK insertion	138 bp insertion	Papp, Sci Rep, 2022	17-0420
**MW837245.1**	UKH47146.1	RPL18 insertion	147 bp insertion	Papp, Sci Rep, 2022	17-0420
**MW837246.1**	UKH47147.1	RdRp insertion	162 bp insertion	Papp, Sci Rep, 2022	17-0420
**MW837247.1**	UKH47148.1	HVR duplication	60 bp insertion	Papp, Sci Rep, 2022	17-0420
**MW837248.1**	UKH47149.1	RPL18 insertion	104 bp insertion	Papp, Sci Rep, 2022	17-0420
**MW837255.1**	UKH47160.1	AHNAK and RdRp insertion	AHNAK insertion: 202 bp+ RdRp insertion: 162	Papp, Sci Rep, 2022	18-0056
**MF444083.1**	AXF47770.1	KIF1B insertion	78 bp insertion	Lhomme, F in Micro, 2020	Hepac-154
**MF444119.1**	AXF47839.1	ZNF787 insertion	138 bp insertion	Lhomme, F in Micro, 2020	Hepac-64
**MF444145.1**	AXF47890.1	RNF 19 A insertion	156 bp insertion	Lhomme, F in Micro, 2020	Hepac-6
**MF444086.1**	information not available	HVR and X-domain insertion	147 bp insertion	Lhomme, F in Micro, 2020	Hepac-12-1
**HQ709170.1**	ADV71352.1	RPS17 insertion	174 bp insertion	Shukla, PNAS, 2011	Hepac-94-2
**MN646696.1**	QHI01839.1	PEBP1 insertion	57 bp insertion	Lhomme, F in Micro, 2020	Hepac-100-2-VHP-6-2
**MN646689.1**	QHI01818.1	GATM insertion	69 bp insertion	Lhomme, F in Micro, 2020	Hepac-100-VHP-6-1
**MN646693.1**	QHI01830.1	RPS17 insertion	108 bp insertion	Lhomme, F in Micro, 2020	Hepac-94-2
**MN646692.1**	QHI01827.1	EEF1A1P13 insertion	57 bp insertion	Lhomme, F in Micro, 2020	Hepac-93-2
**MN646695.1**	QHI01836.1	RNA18SP5 insertion	79 bp insertion	Lhomme, F in Micro, 2020	Hepac-93-3
**MN646694.1**	QHI01833.1	RPL6 insertion	150 bp insertion	Lhomme, F in Micro, 2020	Hepac-26-2
**KJ917704.1**	AIK01907.1	HVR/RdRp duplication	PPR:93 bp + RdRp: 39 bp	Lhomme, J Virol, 2014	S38
**KJ917717.1**	AIK01920.1	HVR duplication	186 bp insertion	Lhomme, J Virol, 2014	S51
**KJ917720.1**	AIK01923.1	TAT insertion	132 bp insertion	Lhomme, J Virol, 2014	S54
**KC166952.1**	AGT96598.1	ITIH2 insertion	75 bp insertion	Lhomme, J Virol, 2014	TLS-09-4-c1
**KC618402.1**	AHC54587.1	HVR/RdRp bipartite insertion	HVR duplication:116 bp+3′-terminal ORF1 region derived:70 bp	Johne, J Viral Hepat, 2013	47832
**JN564006.1**	AEX32827.1	RPS19 insertion /HVR insert	118 bp insertion	Nguyen, Jgen Virol, 2012	LBPR-000379
**MT899358.1**	QOH99407.1	APOC1 insertion	84 bp insertion	Munoz-Chimeno, Micro, 2020	182634MA46_CR3
**MT899359.1**	QOH99408.1	APOC1 insertion	84 bp insertion	Munoz-Chimeno, Micro, 2020	188093MA46_CR3
**OR726753.1- OR726758.1**	information not available	TRIM22 insertion	96 bp insertion	Wißing, Nat Commun, 2024	information not available
**OR726733.1- OR726751.1**	information not available	SERPINA1	108 bp insertion	Wißing, Nat Commun, 2024	information not available
**OR726696.1**	information not available	ALB	168 bp insertion	Wißing, Nat Commun, 2024	information not available
**OR726802.1**	information not available	ALB	168 bp insertion	Wißing, Nat Commun, 2024	information not available
**See Sup** Table 1	information not available	HVR Duplication	144 bp HVR duplication	Wißing, Nat Commun, 2024	information not available

Table of published genomic rearrangements of HEV, including NCBI accession numbers and protein identification (ID), information regarding the type and size of genetic rearrangement, references, and the nomenclature of each recombinant variant.Table 1: Comprehensive Overview of Genomic Rearrangements of HEV. Table of published genomic rearrangements of HEV, including NCBI accession numbers and protein identification (ID), information regarding the type and size of genetic arrangement, references, and the nomenclature of each recombinant variant.

### HVR in acute and chronic HEV infection

HEV represents a significant cause of acute and chronic viral hepatitis, particularly in immunocompromised individuals, where the emergence of specific SNVs in the RdRp has been repeatedly documented^69–71^. Although the emergence of viral variants may contribute to RBV resistance, a causal link to treatment failure has yet to be established.

The HVR, however, is characterized by a higher mutation rate when compared to other genomic regions, and higher frequencies of proline, alanine, and serine residues are reported in this domain^19,21,22,25,110,111,114^. In a recent study, detected insertions in chronically infected patients led to a decrease in proline residues and enrichment of lysine^21,22^. Compared to insertions, deletions are described at lower frequencies in HEV-3, ranging in size from 1 to 63 amino acids^110^. The incorporation of genomic insertions expands the potential for sites that undergo specific post-translational modifications (PTMs), including acetylation, methylation, and ubiquitination, but not phosphorylation or glycosylation^19,21,22,26,110,111^. The proportion of positively charged amino acids was previously reported to increase through host-derived insertions, while duplications derived from HEV itself decreased the proportions of negatively charged amino acids^111^. Additionally, it was also reported that HEV genomic duplications increased the proportion of negatively charged amino acids^110^. Altered local physicochemical properties and PTMs within the HVR may modulate ORF1 domain interactions, which in turn may impact replication complex function and viral RNA synthesis^22,110,111,115^. Studies reporting amino acid compositions of HVR are recovered from chronically infected patients. The exact roles played by protein charge and PTMs, with or without insertions, and their impact on viral replication and the transition to chronicity are currently unclear and require further investigation.

Host genetics can also determine HEV infection outcomes, with approximately 40% of symptomatic HEV patients carrying an inborn error in the IFN system^116,117^. The production of HEV variants with reduced immune recognition may facilitate viral persistence and prolong immune activation, leading to more severe disease courses, especially in HEV-3 clade efg^118^. However, the majority of chronic HEV infections occur in SOT patients, where host adaptive immune pressure is pharmacologically suppressed to prevent organ rejection. The absence of host immune pressure might facilitate the emergence of genomic rearrangements in HVR. Studies of the HVR in recent years have repeatedly suggested that insertions play an important role in modulating the replication capacity of HEV by enhancing or diminishing viral fitness in vitro and in vivo^19,22,25,26^. In the majority of acute HEV infections, the virus typically does not persist for long periods due to effective recognition and clearance by the host adaptive immune system^119^, reducing the likelihood of accumulating mutations or genomic integrations. Due to their transient and often asymptomatic nature, there are only a limited number of studies investigating acutely infected individuals.

Genomic recombination events contribute to the genetic diversity of circulating HEV strains. The first description of genomic rearrangements in acute HEV reports 3/7 chronically infected patients who were retrospectively identified to have acquired HVR insertions during acute infection^111^. This phenomenon was also reported by Biedermann et al., who described acute-to-chronic transition for two HEV-infected patients, with one patient exhibiting emergence of a mix of the HVR and RdRp sequence duplications within the HVR^21^. The study from Muñoz-Chimeno et al. described 90/137 acutely infected patients with a 29 aa duplication in the HVR, identical to 6/7 sequences obtained from swine samples^110^. All were phylogenetically assigned to HEV-3 subtype f. This specific length polymorphism is exclusive to HEV-3f and represents the only insertion identified in swine specimens so far (Fig. 1, asterisk). The majority of HVR duplications of viral origin exhibit variability in both length and the region where the insertion occurs. Furthermore, combinations of duplications from the HVR itself and duplications from other ORF1 domains, including snippets of the RdRp and X domain, are also reported^19–22,86,110,111^ (Fig. 1B). Of note, all reported rearrangements are in frame and do not affect the amino acids downstream of the HVR. An insertion, consisting of a duplicated HVR fragment and an RdRp-derived fragment, enhanced viral replication in a chronically HEV-infected patient with RBV treatment failure^120^. Debing et al. link a K1383N mutation in the RdRp to reduced viral fitness and increased RBV sensitivity, potentially by altered RdRp fidelity, while Y1320H and G1634R mutations compensated by increasing replication. These SNVs were coupled with an 8-amino acid RdRp-derived insertion, which further increased replication efficiency^120^. This suggests HEV adapts through RdRp mutations affecting fidelity and RBV sensitivity, with HVR insertions serving as secondary adaptations to maintain viral persistence under antiviral pressure. Of note, not all patient-derived HVR insertions tested in vitro enhance viral replication^22,26^, highlighting the dependence on insert length, sequence context, and host environment. Together, these data suggest that HVR sequence plasticity likely plays an important role in enhancing HEV replication and potentially impacts pathogenesis, which is so far poorly understood.

**Fig. 2:**
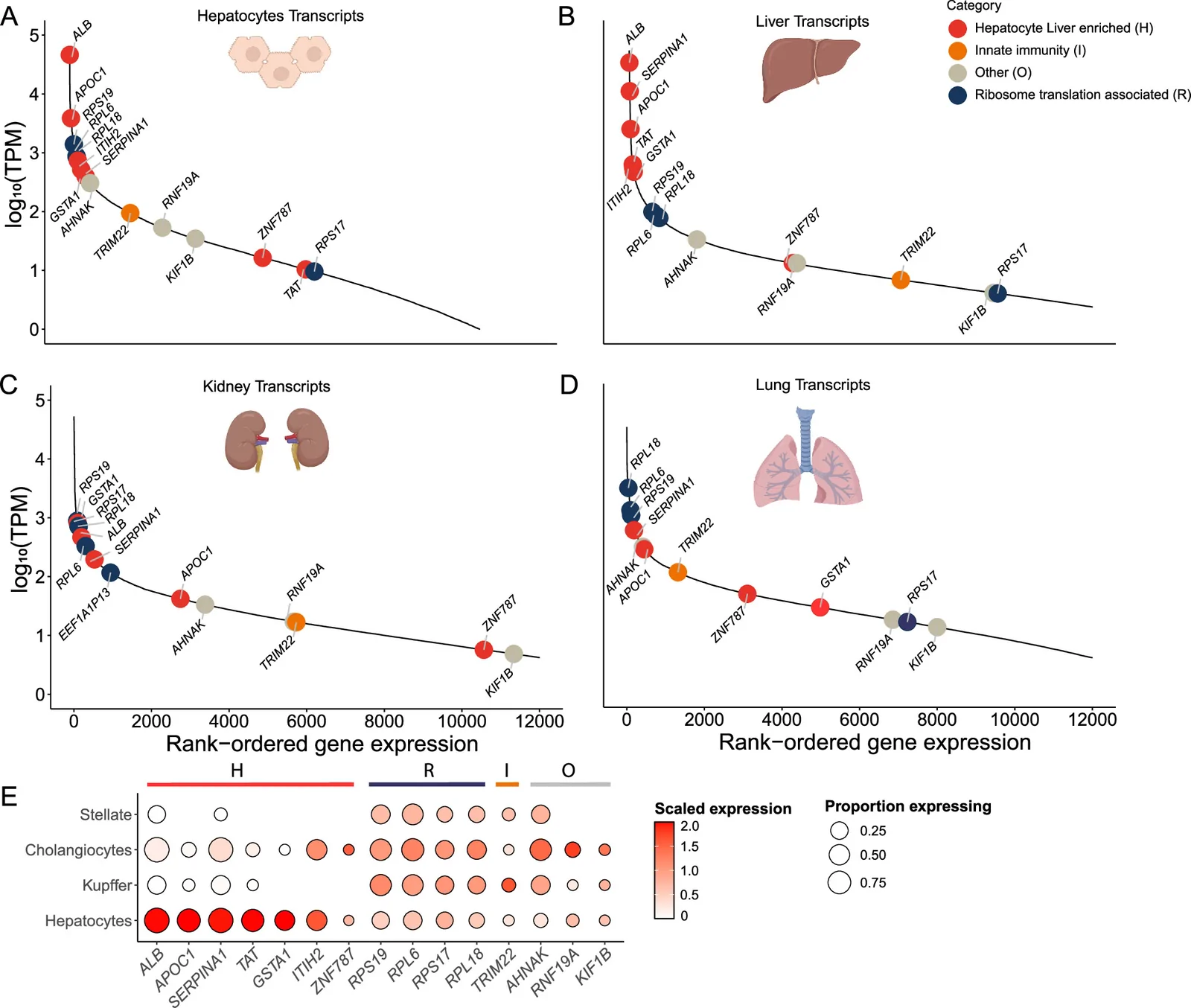
Tissue-specific gene expression profiles of host-derived HVR inserts. **A–D** RNA-seq data were obtained from the NCBI SRA and GEO: for liver (accession numbers SRR8218550, SRR8218566, SRR8218698), kidney (accession numbers SRR9054123, SRR9054125, SRR9054126), lung (accession numbers GSE165192, SRS8082436, SRS8082435, SRS8082437), and hepatocytes (accession numbers GSE166428, SRR13665514, SRR13665520, SRR13665526). Liver, kidney, and lung were selected as they represent the primary site of HEV replication and clinically relevant extrahepatic sites of infection, providing context for the host transcript pool available for potential template-switching events. All data were normalized to transcripts per million (TPM) to allow for comparison within and between conditions. For individual cell or tissue types, TPM values were averaged across donors and log₁₀-transformed. Rank-ordered gene expression in hepatocytes (**A**), liver (**B**), kidney (**C**), and lung (**D**) is represented, illustrating the transcript abundance of HVR-inserted genes across cells and tissues. Genes are categorized into hepatocyte/liver-enriched (red, H), innate immunity-related (orange, I), ribosome/translation-associated (blue, R), and other (gray, O). **E** Data scaling and visualization were performed using the Seurat R package (v5.3.0)^156^, and liver cell type annotations were based on the Human Liver Cell Atlas (GEO: GSE124395) with scaled expression levels extracted accordingly. Scaled expression (color intensity) and proportion of cells expressing specific genes (circle size) across different liver cell types, including hepatocytes, Kupffer cells, cholangiocytes, and stellate cells, are presented. Hepatocyte-specific genes exhibit high expression levels in hepatocytes compared to other liver cell types. In contrast, ribosomal protein-associated genes are expressed constitutively across cell-types. *Parts of figure created in BioRender. Nocke, M. (2026)* https://BioRender.com/g5ambku.

The first reported case of insertion of a human-derived sequence in the HEV HVR was the HEV-3 Kernow-C1-p6 clone, derived from a chronic patient and adapted for growth in cell-culture^85^. This isolate represents the main HEV laboratory strain used for in vitro research. After multiple passages in cell culture, the dominant viral variant contained a fragment of the ribosomal protein S17 (*RPS17*) gene inserted into the HVR, which was shown to enhance viral replication in vitro^85^. This insertion was detected at low frequency in fecal samples from which the original Kernow-C1 strain originated, ruling out the possibility that the insertion was derived from the process of cell-culture adaptation^78,85,121^. This process was also described for the insertion of the ribosomal protein S19 (*RSP19*) gene fragment, derived from a chronic patient’s serum and also exhibiting a growth advantage in cell culture^113^. Subsequently, many other human-derived insertions were detected and characterized in chronically infected patients, generally conferring increased viral fitness. Indeed, in addition to the *RPS17* and *RSP19* fragments, reported HVR insertions of human gene fragments include *TAT*, *ITIH2*, *KIF1B*, *RNF19A*, *ZNF787*, *EEF1A1P13*, *RNA18SP5*, *RPL6*, *GATM*, *PEBP1*, *APOC1*, *RPL18*, *AHNAK*, *GSTA1*, *SERPINA1*, *TRIM22* and *ALB* (Table 1 and Figs. 1, 2)^19–22,85,110,111,113^. In summary, these notions highlight the need to integrate genetic surveillance with clinical data from immunosuppressed or symptomatic HEV patients to systematically link emerging HVR insertions and SNVs to disease course and clinical outcome.

This suggestion is supported by our recent study, wherein HVR inserts derived from a patient with multiple temporally distinct insertions were engineered into the Kernow-C1-p6 clone to characterize their phenotype^22^. Although an effort to investigate the role of altered ORF1 flexibility by HVR rearrangements on viral replication did not yield sufficient evidence in tissue culture using artificially engineered insertions, the influence of favorable proximity of certain ORF1 domains cannot be ruled out^22^. These results highlight the potential pro-viral effect of insertions in the HVR, with enhanced replication. Few rearrangements are also reported to alter the viral sensitivity towards RBV. This phenotype likely relies on several combinatory features like PTM sites, NLS sequences, and possibly higher flexibility within the HVR, as well as improved synergistic interactions with sequence-based determinants outside the HVR. Together, these data suggest that HVR sequence plasticity likely plays an important role in enhancing HEV replication and potentially impacts pathogenesis. However, the molecular mechanisms underlying these virus- and host-sequence “snatching” events remain to be elucidated.

### Potential mechanisms of HEV insertions

Viral surveillance of HEV isolates from swine and acute patients indicates HVR insert-containing variants are not at high frequency in circulating viruses, although there is a clear sampling bias in available sequences. Although limited studies do exist^122,123^, pigs are not routinely sampled, and acute HEV in humans is mostly asymptomatic^124^ and thus not routinely sequenced. Despite this lack, these data imply HVR insert-containing variants may be selectively neutral or deleterious for viral transmission.

Genomic rearrangements are reported in multiple RNA and DNA viruses, occurring by multiple distinct mechanisms and impacting viral evolution, adaptation, and pathogenesis. Across both RNA and DNA viruses, genomic rearrangements are recurrent events that drive prolonged infection and more severe clinical outcomes. Error-prone RNA polymerases and template-switching events repeatedly deposit host-derived fragments, duplications, or chimeric segments into hyper-variable or regulatory regions, yielding variants with higher peak titers, expanded tropism, and diminished immune visibility^125–128^, as seen in Bovine Viral Diarrhea Virus (BVDV), Foot-and-Mouth Disease Virus (FMDV), and Classical Swine Fever Virus (CSFV). Similarly, chronic DNA viruses, e.g., Human Cytomegalovirus (HCMV), exploit recombination, segmental duplication, and even host-genome integration to delete miRNA sites and immunodominant epitopes^129–132^. Irrespective of viral class, these non-random, replication-enhancing changes are consistently enriched under the sustained replication pressure characteristic of immunocompromised hosts, converting self-limiting infections into persistent, therapy-refractory diseases with increased pathogenic potential.

**Fig. 3:**
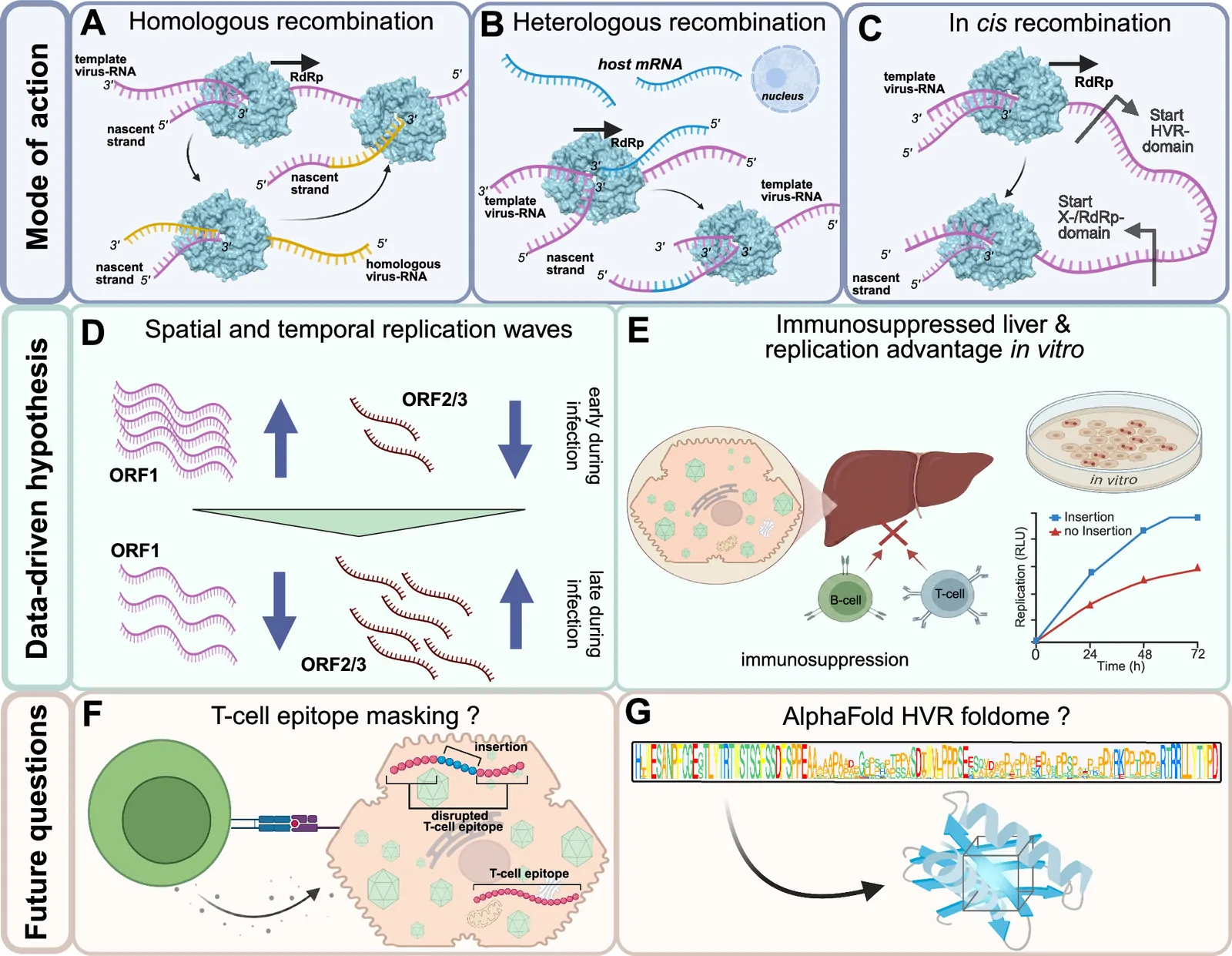
Proposed mechanisms underlying HVR genomic rearrangements in HEV infection. The figure summarizes established mechanisms of action (blue **A**,**B**,**C**), novel hypothesis derived from our sequence analyses in this review and Wißing et al^22^. (green **D**,**E**), and future questions (orange **F**,**G**). **A** Inter-molecular template switching (“homologous recombination”): the RdRp switches between distinct replicating viral RNA molecules in close proximity, generating chimeric viral genomes. **B** Heterologous recombination: RdRp template switching between replicating viral RNAs and host mRNAs in close proximity enables capture of host-derived sequence fragments, with inserted sequences enriched for highly expressed hepatocyte transcripts and/or ribosome-associated RNAs. **C** Intra-molecular RdRp slippage/template switching (“in cis recombination”): RNA folding brings the HVR-encoding domain into close proximity with the X/RdRp-encoding regions on the same molecule, facilitating polymerase slippage and local duplications. **D** Distinct temporal waves of HEV replication: full-length ORF1-encoding genomes and subgenomic ORF2/3-encoding RNAs may replicate in decoupled waves, potentially restricting the genomic regions available for template switching. **E** HVR insert-containing variants proliferate in the immunosuppressed liver with replication advantage, with insertions. **F** Whether the HVR harbors immunodominant T-cell epitopes and whether insertion of host-derived sequences can mediate cellular immune escape requires further in silico, ex vivo, and in vitro characterization. **G** Prediction of the HVR structural landscape (“foldome”) across patient-derived variants using AlphaFold3 may provide insight about common features, flexibility and functions. *Created in BioRender. Nocke, M. (2026)* https://BioRender.com/ui3f5cb.

For HEV HVR insertions and duplications of viral sequences, template switching by the RdRp between distinct replicating viral RNA molecules in close proximity may underlie these “homologous recombination” events, giving rise to chimeric viral genomes with distinct parental RNAs (Fig. 3A). In contrast, chimeric HEV genomes that contain host sequences are derived from progenitor RNA molecules that have different origins: HEV RNAs are produced by the viral RdRp, while host mRNAs are transcribed by RNA polymerase II in the nucleus. After processing and nuclear export, mature host mRNAs are trafficked through the cytosol to the ER for loading onto ribosomes. “Heterologous recombination” between virus and host RNAs via RdRp template switching requires proximity of replicating viral RNAs and host mRNAs (Fig. 3B). While most RNA viruses replicate in the cytosol, enrichment of NLS motifs inserted into HVR suggests nuclear import of pORF1 as well as HEV RNA, may also occur. This observation highlights spatial co-localization of HEV replication sites with host mRNAs, at least in chronically infected cells^22,121^.﻿﻿ Polymerase slippage by the low-fidelity HEV RdRp could underlie duplicated HVR inserts, while inserts from non-adjacent HEV genomic regions are presumably generated by RdRp template switching^125,133^. Of note, HEV genome derived insertions and duplications are restricted to sequences derived from ORF1 encoded domains X and RdRp, which are located downstream of HVR (Fig. 1B). This implies a preference for in cis RNA recombination via RdRp template switching on the same molecule, facilitated by RNA folding during replication which brings the HVR encoding domain into close proximity to the X/RdRp encoding regions (Fig. 3C). Alternatively, in trans template switching could facilitate recombination of sequence fragments between distinct ORF1 encoding template molecules, with stem loop structures located after ORF1 impeding RdRp snatching from the ORF2/3 encoding region. The lack of viral inserts encoding ORF2/3 fragments may also imply distinct temporal waves of HEV replication, with decoupling of replication of ORF1-encoding full-length genomes and production ORF2/3 encoding subgenomic RNAs (Fig. 3D). Whether specific RNA motifs underlie sites of genome recombination or whether viral sequence fragments are snatched from positive-stranded RNA genomes or negative-stranded genomic templates is currently unknown and requires further investigation.

Leveraging NCBI deposited HEV full-length genome sequences from approximately over 650 HEV-3 strains, genome alignment highlights levels of conservation across the genome, confirming HVR is a hotspot for genomic variability (Fig. 1A). Focusing in on HVR encoded amino acids, we generate a sequence logo of the HVR backbone from this alignment, highlighting residue frequencies and cataloging sites of virus- and host-derived insertions (Fig. 1B). HVR insert-containing variants proliferate in immunosuppressed liver by providing a replication advantage^22,26,78,120^. In the absence of inserts, the HVR backbone is clearly enriched for proline residues. Large numbers of proline residues in a protein domain would have structural and functional consequences. Prolines are known to disrupt the formation of regular secondary structures like alpha helices and beta strands, and also promote the formation of turns and loops, leading to more flexible or disordered regions within the protein. Proline-rich regions are also known to be involved in protein-protein interactions and are found in various domains like collagens, transmembrane proteins, and SH3 domains^134^. The exact contribution of HVR proline enrichment to replication enhancement and persistence remains unclear and requires further investigation.

We summarize all previously described HEV-3 insertions and map their locations within the central HVR (Fig. 1B). In addition, we identified a previously undescribed 49-amino-acid human-derived insertion, a partial fragment of the vacuolar protein sorting-associated protein 4 A (*VPS4A*) protein, sourced from a blood sample of a HEV-3f-infected patient in France^37^. Human-derived insertions encode fragments of diverse host proteins (Fig. 1B), although close inspection of underlying nucleic acid sequences at selected HVR insertion hot-spots indicates there are no enriched nucleotide motifs associated with host-virus integration sites (Fig. 1C). However, the integration process is not completely random, with host derived inserts enriched for specific biological processes within the host-cell or highly expressed in specific cell-types (Fig. 2). Leveraging publicly available RNA-seq data from primary human hepatocytes (Fig. 2A) and distinct human tissues including liver, kidney and lung (Fig. 2B–D), we investigated the intrinsic transcript abundance of HVR integrated host genes in these cells/tissues. HEV is primarily a hepatotropic virus, although extrahepatic manifestations in non-hepatic liver cells, with associated HEV infection and replication, are also described^77,135–141^. In line with this, we noted three major subsets of HVR integrated host genes: either genes had high intrinsic expression in hepatocytes/liver (*ALB*, *TAT*, *ITIH2*, *SERPINA1*, *GSTA1*, *APOC1*, *GATM*, *VPS4A*), are known interferon-stimulated genes and upregulated in the context of HEV infection (*TRIM22*), or encode structural components of the ribosome or are associated with RNA binding and translation (*AHNAK*, *RPL6*, *RPS17*, *RPL18*, *RPS19*, *RNA18SP5*, *ZNF787*, *PEBP1*, *EEF1A1P13*) (Table 1). Using liver atlas deposited single-cell RNA-seq (scRNA-seq) data we confirm strong expression of hepatocyte transcripts specifically in hepatocytes, while ribosomal/translation specific transcripts were constitutively expressed across all cell types, as expected (Fig. 2E). These data imply a bias toward insertion of transcripts that are highly expressed in hepatocytes, consistent with their role as the major target cell for HEV replication and associated pathology. Furthermore, enrichment of ribosomal/translation associated genes implies potential co-localization of HEV replication sites with ribosomes, where these transcripts would be enriched within the cell. Together, these data support the hypothesis that the distribution of host HVR inserts is not random, and rather directly linked to both transcript abundance and localization within infected cells. Most likely, the in -frame insertion into the HVR confers the replication advantage, rather than the physiological function of the host proteins from which the inserts are derived.

Immunosuppressants used in SOT recipients target B- and T-cell arms of the adaptive immune system^142^ so impaired humoral and cellular targeting contribute to the progression of HEV to chronicity and the emergence of HVR inserts. Therefore, the mechanism that underlies the accumulation of HEV genomic insertions likely involves conferring a replication advantage, but only in the absence of adaptive immune pressure. However, when considering in vitro models to study HEV, there is no selection pressure applied by the adaptive immune system along with some aspects of innate immunity^143^ (Fig. 3E).

HVR inserts are likely selectively deleterious in the face of humoral and cellular targeting, which would explain their low frequency in circulating strains. Due to HVR’s location in the non-structural proteins, which are not associated with virions, it is unlikely that inserts emerge due to impaired antibody targeting. In contrast, viral replication complex proteins often contain immunodominant T-cell epitopes, and these proteins are highly expressed in infected cells. Whether the HVR contains immunodominant T-cell epitopes, and if so, whether cellular immune escape can be mediated by insertion of host sequences into T-cell epitopes requires further bioinformatic, ex vivo, and in vitro characterization (Fig. 3F). The insertion of virus-host sequence snippets contributes to the highly variable and intrinsically disordered tertiary structure of the HVR protein domain^144^, rendering cryo-EM or crystal structures of HEV HVR challenging. Sampling the ‘foldome’ of HVR variants in 3-dimensional sequence space using artificial intelligence-based structural predictions (Alphafold3) may provide insights into the evolution and the biological function(s) of this domain^145,146^ (Fig. 3G).

### Early detection of genomic rearrangements in HEV infection

While the detection of HEV in diagnostic laboratories and clinics is routine, predictors of disease severity and potential chronicity are lacking. Although immunosuppression strongly influences the course of infection, viral factors such as SNVs or HVR rearrangements cannot be excluded, especially during RBV treatment failure. Elucidating the dynamics of HEV genome plasticity during chronic infection helps to identify viral determinants for chronicity and therapy failure and consequently inform the development of novel diagnostic markers to improve clinical management.

An HEV infection represents a considerable risk for immunosuppressed patients, which creates a significant challenge in clinical settings. The late detection and lack of monitoring of an HEV infection can result in the development of progressive liver fibrosis and cirrhosis^43,44,147^. Therefore, it is of the utmost importance to detect the infection at an early stage to facilitate the prompt and effective administration of antiviral treatment. Diagnosis of HEV infection is often under-reported because symptoms overlap with other forms of hepatitis. Serological testing is required for accurate diagnosis, but these tests are not always readily available, especially in resource-limited settings^148^. These combined diagnostic approaches are becoming increasingly prevalent and have been implemented in numerous developed countries, including Germany^149^, to identify low viral loads in the early stages of an acute infection in blood donors^124,150^. Awareness, early diagnosis, and appropriate management are key to mitigating HEV’s impact in clinical settings. While detection through RT-qPCR is well established for known sequences to quantify specific insertions, it has limitations in terms of size, resolution, and the ability to analyze unknown or complex insertions. As demonstrated in the studies by Lhomme et al. and Wißing et al.^22,111^, multiple insertions could be characterized in samples from one chronically HEV-infected patient, including both novel and previously described host fragments. Sequencing HEV diversity within the host is currently insufficient and only reported in a few limited studies. Together, this highlights the need for larger cohort studies together with robust and reproducible methods for sequencing HEV genomes to detect HVR genomic rearrangements, which could serve as a biomarker for chronicity and a potential determinant for severity.

Detailed characterization of insertions in HEV-infected individuals relies on sequencing technologies, which are crucial for developing personalized antiviral strategies. Sanger sequencing has traditionally been used for the HVR due to its ability to handle unpredictable insertion lengths and provide long, accurate reads^151^. However, this method can be labor-intensive as the HEV genome or HVR must first be amplified and cloned; multiple individual clones must be sequenced independently to give a snapshot of the sample diversity, which may not be completely captured. In contrast, next-generation sequencing (NGS) platforms offer multiple advantages^152–154^, most notably capturing all the sample genetic diversity in a single experiment. Illumina short-read sequencing strategies are known for high accuracy, precision, and scalability for larger projects using high-capacity instruments (e.g., NovaSeq platform) that allow sequencing of up to 600 bases. However, one disadvantage of short-read approaches is that linkage between polymorphisms is difficult to assign, which is an important consideration for evolutionary studies of rapidly evolving RNA viruses. Addressing this issue, Oxford Nanopore Technology (ONT) offers ultra-long reads (up to 2 million bases), real-time data streaming, and portability, but with a higher error rate^153^. Similarly, the PacBio system also produces ultra-long reads, with a significant advantage in accuracy, particularly in high-complexity regions, although disadvantages include costs and scalability for high-throughput applications. Together, these NGS platforms will be critical for future studies of HEV genomic rearrangements, particularly for understanding chronicity, immune escape, and antiviral resistance. However, current bioinformatic tools, while effective for SNV analysis, remain poorly adapted for insertion detection, presenting an ongoing challenge for comprehensive HEV genomic characterization. Past studies demonstrate that specific SNVs combined with HVR insertions enhance viral replication, drive antiviral treatment failure, and promote chronicity in HEV infections^21,22,120^. These findings underscore the need to fully characterize the spectrum of genomic rearrangements that occur during infection. The limits of Sanger sequencing highlight that a switch to NGS sequencing is long overdue. NGS enables full capture of the complexity of multiple simultaneous HVR insertions, together with associated polymorphisms, apparent in HEV populations sampled from individual patients. At present, there is a lack of reliable and efficient identification algorithms that quantify the occurrence of larger genomic fragments in the HEV genome. Such tools are necessary for the monitoring of infection courses and to inform therapeutic interventions.

Given these complex mechanisms, it is crucial to test for HEV in cases of symptomatic acute hepatitis. Screening for HEV RNA with subsequent sequencing will provide valuable insights to identify insertions that support chronicity, by utilizing NGS combined with diagnostic bioinformatics tools. Therefore, the detection of genomic rearrangements and increasing the availability of a larger dataset of cohorts will be essential in guiding future treatment strategies for chronic HEV.

### Outlook

Like many RNA viruses, HEV undergoes genetic alterations to adapt and gain evolutionary advantages rapidly across changing environmental conditions, including antiviral therapy. The absence of proofreading mechanisms in the RdRp of RNA viruses is a contributing factor to the high mutation rate for HEV, as evidenced by studies of small cohorts of immunocompromised patients that confirmed the presence of HVR mutations coupled with genomic rearrangements, which together expanded the diversity of the virus’s genetic landscape. This expanded genetic diversity seems to offer a selective advantage in certain settings, enabling HEV to adapt to a broad spectrum of hosts, persist in immunocompromised patients over extended periods, and facilitate cross-species transmission. The precise mechanisms through which genomic rearrangements and host sequence snatching events provide evolutionary advantages leading to immune evasion, treatment failure, and viral persistence require further research, combined with improved surveillance. The study of SNVs, especially in the viral polymerase in HEV-infected patients, is an active area of research, and the observed variations in SNVs between genotypes and the patterns of mutations may be influenced by host-specific adaptations and the use of antiviral treatments enabling insertions. Combining continued population monitoring at national and global levels will enable improved cataloging of HVR insertions, their association with specific SNV signatures, and their correlation with clinical outcomes. However, current publicly available genomic data may suffer from sampling biases, as certain regions and patient populations (e.g., low-resource settings, low surveillance, or underrepresented genotypes and subtypes, acute infections) are often underrepresented. In the past, greater reliance was placed on biochemical assays due to less advanced technology. However, with the advent of NGS and improved cell culture systems, rare variants that arise in the host are now being identified before being introduced into cell culture systems. Despite this, limited investigation regarding HEV genomic rearrangements hinders progress. To address this knowledge gap, further research and study of the HEV genome in vitro is necessary, with the aim of fostering a more comprehensive understanding of the role and importance of HVR in HEV replication. Notably, NGS techniques are currently underutilized in clinical practice, despite their potential for early detection of viral infections. The application of NGS and bioinformatics tools enables researchers to gain insights into the evolutionary dynamics of viruses, facilitating the identification of critical mutations that may influence disease progression and persistence. Further research, accompanied by improvements in sequencing techniques and the development of automated, freely available identification software, and coupled with phenotypic data, is necessary to fully capture the evolutionary advantages conferred by genomic rearrangement in HEV HVR to aid clinicians in personalized approaches to treatment options.

## Supplementary information

Supplement Material

## Data Availability

Data analyzed in this review article were accessed at repositories as indicated in the figure legends and respective references.
