# Supplementary material for: Hepatitis E virus genome plasticity: a marker of chronicity and therapy failure?
Source: Commun Biol. 2026 Oct 22;9:1388. doi: 10.1038/s42003-026-11055-5 (PMC13691058; doi:10.1038/s42003-026-11055-5)
Supplement: Supplementary file 1 [file 42003_2026_11055_MOESM1_ESM.pdf]

Supplementary Information to

Hepatitis E Virus Genome Plasticity: A Marker of Chronicity and Therapy Failure?

Supplementary Table 1: NCBI accession numbers for HVR duplication insertions accompanying table 1.

OR726668.1
OR726669.1
OR726670.1
OR726671.1
OR726677.1
OR726682.1
OR726684.1
OR726686.1
OR726687.1
OR726688.1
OR726689.1
OR726690.1
OR726691.1
OR726692.1
OR726693.1
OR726694.1
OR726695.1
OR726697.1
OR726699.1
OR726700.1
OR726703.1
OR726704.1
OR726706.1
OR726707.1
OR726708.1
OR726709.1
OR726710.1
OR726711.1
OR726715.1
OR726716.1
OR726720.1
OR726721.1
OR726722.1
OR726723.1
OR726724.1
OR726725.1
OR726727.1
OR726759.1
OR726761.1
OR726781.1
OR726788.1
OR726789.1
OR726790.1
OR726791.1
OR726792.1
OR726793.1
OR726794.1
OR726795.1
OR726796.1
OR726797.1
OR726798.1
OR726803.1
OR726804.1
OR726805.1
OR726806.1
OR726809.1
OR726810.1
OR726811.1
OR726812.1
OR726815.1
OR726816.1
OR726817.1
OR726818.1
OR726819.1
OR726820.1
OR726821.1
OR726822.1
OR726824.1
OR726826.1
OR726828.1
OR726829.1
OR726831.1
OR726833.1
OR726834.1
OR726835.1
OR726837.1
OR726838.1
OR726840.1

OR726762.1
OR726763.1
OR726765.1
OR726767.1
OR726768.1
OR726769.1
OR726770.1
OR726772.1
OR726778.1
OR726779.1
OR726780.1
OR726841.1
OR726842.1
OR726844.1
OR726846.1
OR726847.1
OR726848.1
